# Chitosan Ascorbate Nanoparticles for the Vaginal Delivery of Antibiotic Drugs in Atrophic Vaginitis

**DOI:** 10.3390/md15100319

**Published:** 2017-10-19

**Authors:** Silvia Rossi, Barbara Vigani, Antonella Puccio, Maria Cristina Bonferoni, Giuseppina Sandri, Franca Ferrari

**Affiliations:** Department of Drug Sciences, University of Pavia, Viale Taramelli 12, 27100 Pavia, Italy; barbara.vigani@unipv.it (B.V.); antonella.puccio@unipv.it (A.P.); cbonferoni@unipv.it (M.C.B.); giuseppina.sandri@unipv.it (G.S.); franca.ferrari@unipv.it (F.F.)

**Keywords:** chitosan ascorbate, nanoparticles, vaginal administration, fast dissolving polymeric matrix, wound healing

## Abstract

The aim of the present work was the development of chitosan ascorbate nanoparticles (CSA NPs) loaded into a fast-dissolving matrix for the delivery of antibiotic drugs in the treatment of atrophic vaginitis. CSA NPs loaded with amoxicillin trihydrate (AX) were obtained by ionotropic gelation in the presence of pentasodium tripolyphosphate (TPP). Different CSA:TPP and CSA:AX weight ratios were considered and their influence on the particle size, polydispersion index and production yield were investigated. CSA NPs were characterized for mucoadhesive, wound healing and antimicrobial properties. Subsequently, CSA NPs were loaded in polymeric matrices, whose composition was optimized using a DoE (Design of Experiments) approach (simplex centroid design). Matrices were obtained by freeze-drying aqueous solutions of three hydrophilic excipients, polyvinylpirrolidone, mannitol and glycin. They should possess a mechanical resistance suitable for the administration into the vaginal cavity and should readily dissolve in the vaginal fluid. In addition to antioxidant properties, due to the presence of ascorbic acid, CSA NPs showed in vitro mucoadhesive, wound healing and antimicrobial properties. In particular, nanoparticles were characterized by an improved antimicrobial activity with respect to a chitosan solution, prepared at the same concentration. The optimized matrix was characterized by mechanical resistance and by the fast release in simulated vaginal fluid of nanoparticles characterized by unchanged size.

## 1. Introduction

During the last decades, therapies targeting cervical cancer have been considerably improved. Surgery and radiotherapy (RT) represent the main common therapeutic approaches in cervical cancer [1]. In particular, pelvic RT plus intracavitary RT is usually given in early-stage disease, while pelvic RT or whole abdomen irradiation in advanced disease [2,3]. Among the side effects associated with RT, vaginal atrophy is the most important and common one [1]. Vaginal atrophy, also called atrophic vaginitis, is an inflammation of the vaginal cavity, associated to relevant symptoms, such as itching, burning and dyspareunia, which heavily affect the quality of women’s lives. The anatomical and physiological alterations of the vaginal mucosa and the weakening of the immune system related to cancer therapies can result in mucosa lesions and bacterial infections [4,5].

The risk of infections can be reduced by the concomitant use of anti-infective drugs and wound repairing strategies, which involve the use of bioactive compounds able to improve tissue regeneration.

In the last decades, the scientific community has invested much effort in the study of biomaterials capable of interacting with the tissue components taking part to the healing process. Among these, chitosan has been described for its anti-infective, radical scavenging and wound healing properties [6,7,8,9,10,11,12]. Several mechanisms are reported in the literature to explain chitosan activity on wound healing. It is recognized that chitosan acts as chemo-attractant for neutrophils and stimulates granulation tissue formation or re-epithelization [9]. Moreover, chito-oligomers work as bricks in the synthesis of hyaluronan, that, in turn, promotes cell motility, adhesion and proliferation, thus playing an important role in tissue repair. It has been demonstrated that chitosan promotes regeneration, shows a stimulatory effect on macrophages and inhibits metalloproteinases [9].

Furthermore, chitosan exhibits antioxidant properties, which aid in moderating inflammation, and mucoadhesive potential that allows prolonged drug permanence into the vaginal cavity [13,14].

In a previous work of ours, a new salt of chitosan ascorbate (CSA) was selected among other polymers as the most promising candidate for the development of mucoadhesive systems intended for the treatment of mucositis [11]. In particular, it was characterized by: (i) mucoadhesive properties; (ii) antimicrobial activity against *Escherichia coli* and *Staphylococcus aureus*; (iii) increased antioxidant properties with respect to chitosan hydrochloride due to the salification with ascorbic acid and iv) in vitro ability to enhance fibroblast proliferation. Recently, in a previous work of ours, CSA nanoparticles (NPs) were developed for the systemic vaginal delivery of peptides [15].

So far to the best of our knowledge, no papers have been published on the use of CSA for the preparation of NPs with the aim of promoting vaginal atrophy healing, thanks to CSA bioactive properties. Such NPs were intended for the vaginal delivery of an antibiotic drug. Regarding NP administration, a key issue concerns the choice of vehicle that should allow NPs to be easily administered and release without modifying their features [15].

Given these premises, the aim of the present work was the development of CSA NPs for the delivery of a model antibiotic drug, amoxicillin trihydrate (AX), into the vaginal cavity. CSA NPs were prepared by ionotropic gelation in the presence of pentasodium tripolyphosphate (TPP). In particular, the effect of the polymer/TPP ratio on NP size and production yield was investigated. The optimal ratio between CSA and AX was also studied; the association efficiency and loading capacity were specifically evaluated. Subsequently, the NP capability to interact with vaginal mucosa was investigated by means of a turbidimetric method using porcine gastric mucin as a biological substrate [16,17]. The physical stability of an aqueous NP suspension was also investigated upon storage at 4 °C. Moreover, NPs were characterized for antimicrobial properties against *Enterococcus hirae* and *Streptococcus pyogenes* in comparison with a CSA solution. The in vitro wound healing properties of NPs were also investigated on human fibroblasts. In order to permit their administration into the vaginal cavity, NPs were loaded in a freeze-dried matrix based on polyvinylpyrrolidone (PVP), mannitol (MNT) and glycin (GLY). The matrix composition was optimized by means of a DoE approach [18,19,20,21]; a simplex centroid design was used. The optimized matrix was characterized in terms of the mechanical resistance that should be suitable for withstanding stresses occurring during packaging and administration; additionally, the optimized matrix should exhibit the capability to ready dissolve upon administration, releasing NPs.

## 2. Results and Discussion

### 2.1. Characterization of Unloaded NPs (Blank)

In the first phase of the research, experiments were performed to identify optimal operative conditions for NP production. These experiments mixed aqueous solutions containing CSA and TPP in different ratios. Particles were obtained by ionotropic gelation, a technique largely employed for the encapsulation of molecules soluble in aqueous media. The main advantage of this method is that it avoids the use of high energy and chemical reagents, which could degrade the loaded drug [22].

The size, polydispersion index (P.I.) and yield (%) values of blank NPs, prepared according to different CSA/TPP *w*/*w* ratios and obtained by means of single shot mixing technique are reported in Table 1.

Recently, some authors investigated the influence of the method by which TPP is added to chitosan salt solution on average particle size [23]. Specifically they compared single shot mixing, dropwise addition and the dilution technique, where polymer and TPP are dissolved in high ionic strength conditions (in order to inhibit gelation) and then diluted to lower ionic strength. Among these methods, dilution yielded the lowest particle size at high ionic strength conditions. Since the mixing procedure did not significantly affect NP size at low ionic strength conditions (i.e., in the absence of salts other than TPP), as in our case, we chose to prepare NPs by means of a single shot mixing technique. The NPs had a diameter ranging into 200–260 nm intervals, in accordance with the size described in the literature for NPs obtained by ionotropic gelation between chitosan and TPP [15,23]. The polydispersion index (P.I.) was in the range of 0.1–0.3, suggesting that the sample is quite homogeneous. The low variability of both size and P.I. values (calculated on NPs obtained by three different preparations) pointed out the reproducibility of the method.

Table 1 shows that the NP size becomes smaller and the production yield increases with a decreasing CSA/TPP ratio (i.e., an increasing TPP amount). These results are in accordance with what is reported in the literature [15,24]; a high TPP amount leads to a more crosslinked and compact NP structure. This affects particle swelling, especially at low ionic strength, and results in smaller particle size. The increase in yield (%) on the increasing TPP amount indicates that the stoichiometry of chitosan and TPP interaction has not been reached.

### 2.2. Characterization of AX Loaded Nanoparticles

#### 2.2.1. Size and Production Yield

Regardless of the CSA/AX *w*/*w* ratio, AX loading produced only slight variations in NP size, in the range of 210–270 nm (Table 2). Moreover, it can be observed that the loaded NPs were characterized by higher production yield in comparison with blank NPs (Table 1). This can be due to AX acidic properties and, thus, to the occurrence of chitosan–AX interaction [25]. Among the CSA/TPP ratios considered, the highest yield (%) values were observed for 3:1 CSA/TPP values. For such ratio, an increasing in yield (%) values is observed on increasing the CSA/AX weight ratio.

#### 2.2.2. Association Efficiency (AE) and Loading Capacity (LC)

**Figure 1:**
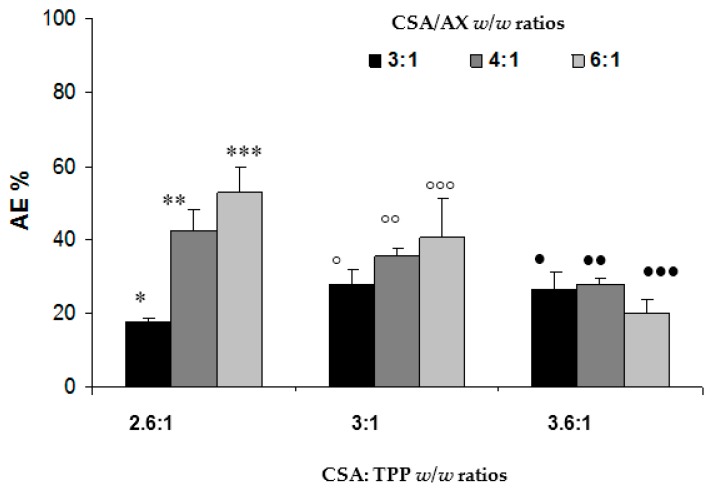
Association efficiency (AE) values obtained for NPs prepared according to different CSA/TPP and CSA/AX *w*/*w* ratios (mean values ± s.e.; *n* = 3). One-way analysis of variance (ANOVA), multiple range test (*p* < 0.05): * versus **; * versus ***; *** versus •••; °°° versus •••.

In Figure 1, the AE values observed for NPs prepared according to different CSA/TPP and CSA/AX *w*/*w* ratios are reported. For 3:1 and 3.6:1 CSA/TPP ratios, the AX amount does not significantly affect the AE values (one-way analysis of variance (ANOVA)). On the contrary, for the 2.6:1 CSA/TPP ratio an increase in AE values was observed on decreasing the AX amount.

These results can be justified by the occurrence of an interaction between AX and chitosan. Lowering the polymer amount available for the interaction, the AE value becomes smaller. Finally, at the same CSA/AX ratio, no significant differences were observed between the AE values obtained for different CSA/TPP ratios, with the exception of the 6:1 CSA/AX ratio. In this case, significantly different AE values were observed between CSA/TPP 2.6:1 *w*/*w* and 3.6:1 *w*/*w* ratios and between 3:1 and 3.6:1 *w*/*w* ratios. A high amount of CS with respect to TPP (ratios > 3:1) produced a reduction in the AE value, indicating that slightly packed NPs are not able to entrap AX.

**Figure 2:**
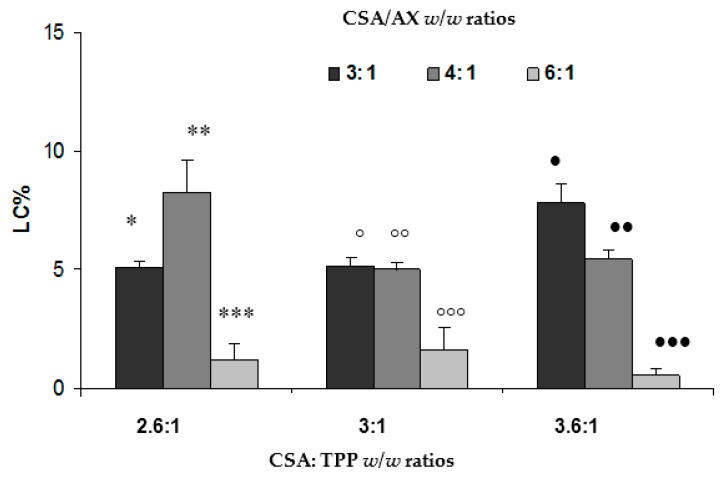
Loading capacity (LC) values obtained for NPs prepared according to different CSA/TPP and CSA/AX *w*/*w* ratios (mean values ± s.e.; *n* = 3). one-way ANOVA, multiple range test (*p* < 0.05): * vs. ***; ** vs. ***; ° vs. °°°; °° vs. °°°; • vs. •••; • vs. ••; •• vs. •••; * vs. •; ° vs. •.

In Figure 2, the LC values observed for NPs prepared according to different CSA/TPP and CSA/AX *w*/*w* ratios are reported. NP LC increases on decreasing the CSA/AX *w*/*w* ratio from six to four. A further decrease of this ratio did not produce any significant changes in LC values, except when the 3.6:1 *w*/*w* ratio was considered.

On the basis of the results so far obtained, NPs prepared according to 2.6:1 *w*/*w* CSA/TPP and 4:1 *w*/*w* CSA/AX were chosen for the continuation of the work.

#### 2.2.3. Stability Test

**Figure 3:**
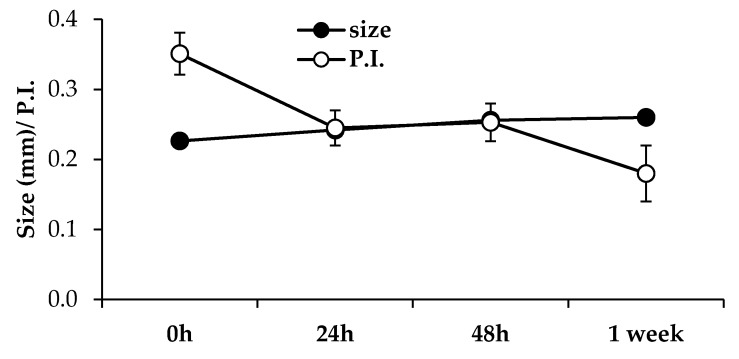
Size and P.I. of NP suspension stored for different times at 4 °C in refrigerator (mean values ± s.e.; *n* = 3).

The stability of nanoparticles is a critical issue, since one of the major problems encountered with colloidal systems is their tendency to aggregate upon storage. In Figure 3, the size and P.I. of the NP (6:1 *w*/*w* CSA/TPP and 4:1 *w*/*w* CSA/AX) suspension, stored for different times at 4 °C, are reported. Storage for up to one week produced a slight increase in size, even if not statistically significant, and a marked decrease in P.I. Such results pointed out that NPs do not aggregate in aqueous suspension. As expected, NPs show a positive Z potential (34.9 ± 1.35 mV) that hinders aggregation.

#### 2.2.4. Mucoadhesion Properties

**Figure 4:**
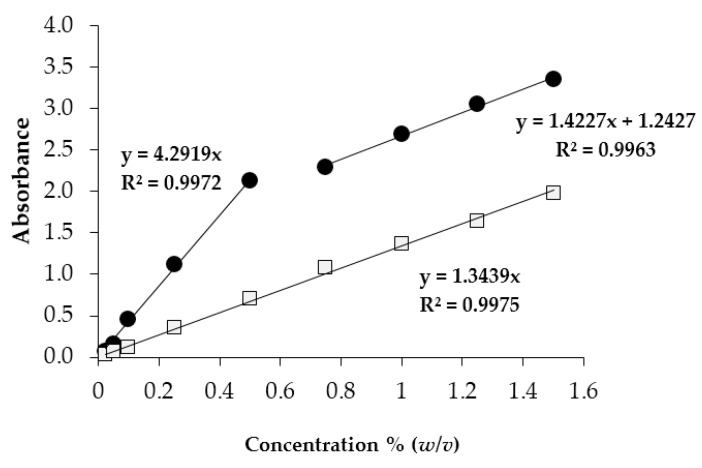
Absorbance values measured for mucin dispersion at pH 4.5 (open squares) and NP–mucin mixtures (closed circles).

The assessment of the NP mucoadhesion properties by means of turbidimetric measurements is based on the hypothesis that the mucoadhesive interaction between the sample and mucin results in mucin adsorption onto NPs, and in their slight aggregation, which can be detected as an increase in turbidimetry [17]. Figure 4 reports the results of turbidimetric measurements. The increase in mucin concentration produces, as expected, an increase in turbidity in mucin dispersion. The trend of the experimental data (plotted as absorbance vs. mucin concentration) is fitted by a linear equation reported in Figure 4. Considering NP–mucin mixtures, at a mucin concentration up to 0.8% (*w*/*v*), the slope of the straight line that fits the experimental data is higher than that obtained for mucin dispersions. This result means that, when NPs are mixed with mucin, a synergic increase in turbidity occurs, suggesting that the mucoadhesive interaction results in NP aggregation. At a mucin concentration above 0.8% (*w*/*v*), the data are interpolated by a straight line that has a slope similar to that obtained for mucin dispersion. This is attributable to the saturation of NP–mucin interactions. In this scenario, the turbidity increase is only due to an increase in mucin concentration.

#### 2.2.5. Antimicrobial Activity Measurements

Table 3 reports antibacterial activity, as a minimum inhibitory concentration (MIC), against *E. hirae* and *S. pyogenes* of blank and loaded NPs (CSA/TPP 2.6:1; CSA/AX 4:1) in comparison with a CSA aqueous solution prepared at the same concentration (0.135% *w*/*w*) employed for NP preparation. MIC is expressed as % *w*/*v* CS.

It can be observed that the polymer in free form has a less inhibitory effect than NPs. Several mechanisms have been proposed to explain chitosan antimicrobial properties, such as: (i) chelation of trace elements or essential nutrients, required for bacteria growth; and (ii) interaction with anionic groups on the cell surface and the formation of polyelectrolyte complexes with bacterial surface compounds, thereby forming an impermeable layer around the cell, which prevents the intracellular transport of essential nutrients [26,27]. According to some authors, the antimicrobial properties of CS are particularly effective against Gram-positive bacteria, which are characterized by a negatively charged surface, due to the presence of lipopolysaccharides containing phosphate and pyrophosphate groups [28]. This morphological feature promotes the attachment of CS cationic chains through effective electrostatic interactions.

The higher NP antibacterial activity with respect to free chitosan depends on the peculiar properties of nanosystems. Polycationic chitosan NPs with a high surface charge density are able to interact with bacteria cells to a greater extent than free polymer. NPs provide higher affinity with bacteria cells for a quantum–size effect. It has been recently reported in the literature that NPs can penetrate bacteria, disrupting the cell membrane more than bigger particles [29]. As expected, the presence of AX improves antibacterial activity.

#### 2.2.6. Proliferation Assay

**Figure 5:**
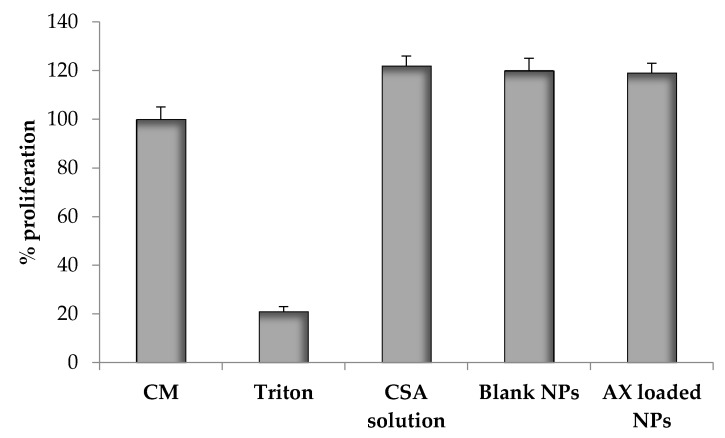
Proliferation (%) of normal human dermal fibroblasts (NHDF) after incubation (24 h; 37 °C) with complete medium (CM), Triton X-100, CSA aqueous solution (0.135% *w*/*w*), blank NPs and AX-loaded NPs (mean values ± s.e.; *n* = 8).

In Figure 5, cell proliferation results related to blank and loaded NPs are reported. Despite dilution in medium without serum, all samples, with the exception of Triton X-100 (negative control), promote cell proliferation as the positive control (CM). These results confirm the positive effect of CSA on fibroblast proliferation. Moreover, it is demonstrated that the samples containing the polymer (blank and loaded NPs and the CSA solution 0.135% *w*/*w*) promote cell proliferation with no significant differences (ANOVA one-way), suggesting that neither the NP preparation method nor the presence of AX alter the intrinsic capability of CSA to enhance cell proliferation.

#### 2.2.7. Wound Healing Assay

**Figure 6:**
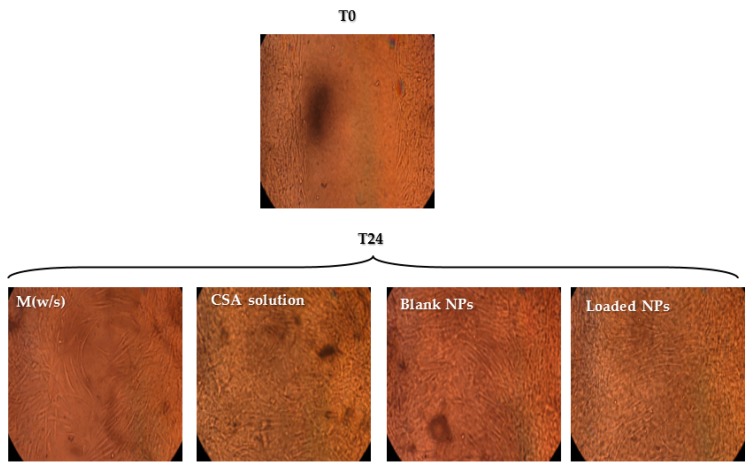
Microphotographs of fibroblasts after 24 h (T24) contact with different samples: medium without serum (M w/s), CSA solution, blank NPs, loaded NPs. At time zero (T0), a cell-free gap is visible for all the samples considered.

Fibroblasts have a crucial role in mucosal healing; after a wound occurs, activated fibroblasts appear in response to injury. Moreover, fibroblasts migrate to the wounded area, secreting collagenases, proteases and extracellular matrix components, which contribute to the reconstruction of damaged mucosa [30]. Figure 6 shows the microphotographs of fibroblasts after 24 h (T24) contact with: (a) medium without serum (M (w/s)); (b) CSA solution; (c) blank NPs (d) AX-loaded NPs. At time zero (T0), cell-free gap is visible for all the samples considered. After 24 h contact (T24), a gap is still evident for M (w/s), whereas in the presence of NPs (blank or loaded) and CSA solution, fibroblasts have grown up to confluence, indicating wound healing properties. Various mechanisms are reported in the literature for chitosan activity on wound healing. As already mentioned in the introduction, chitosan works as chemo-attractant agent for neutrophils and promotes granulation tissue formation or re-epithelization [9,30]. Moreover, chitosan promotes dermal regeneration, has a stimulatory effect on macrophages and inhibits metalloproteinases. Analogously to the antimicrobial effect, the improvement in wound healing can be due to the large surface area of NPs, responsible for a deeper interaction with fibroblasts.

### 2.3. Characterization of Fast Dissolving Matrices

#### 2.3.1. Mechanical Resistance of Matrices

**Figure 7:**
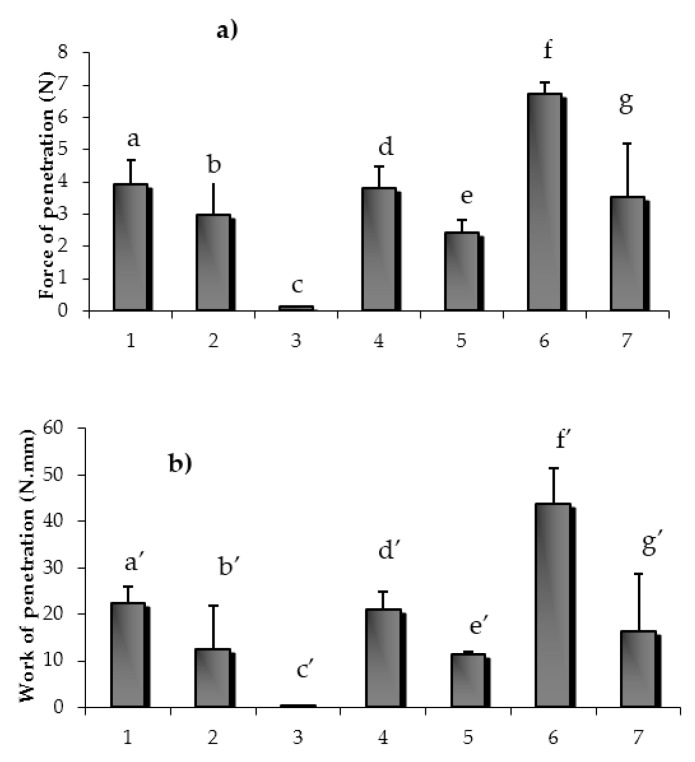
Mechanical properties, expressed as penetration force (**a**) and penetration work (**b**) of the different matrices of the experimental design (mean values ± s.e.; *n* = 3). One-way ANOVA, multiple range test (*p* < 0.05): a vs. c/f/e; b vs. c/f; c vs. d/e/f/g; a’ vs. c’/f’/e;’ b’ vs. c’/’f; c’ vs. d’/e’/f’; d’ vs. e’.

In Figure 7a,b, the mechanical properties, expressed as penetration force (a) and penetration work (b), of the different matrices of the experimental design are reported. The highest mechanical resistance, indicated by the highest values of both penetration force and work, was observed for matrix six, based on PVP 1.5% *w*/*w* and GLY 2.5% *w*/*w*, while matrix three, based only on GLY (5% *w*/*w*), showed the lowest values of mechanical resistance. As expected, PVP is the excipient playing the main role in the matrix mechanical properties. Better performance was obtained when PVP was mixed with GLY with respect to MNT.

Nevertheless, all the matrices, with the exception of that based on pure GLY, maintained integrity when handled. They possess a mechanical resistance adequate to their use, which involves a mild manipulation.

#### 2.3.2. Dissolution Time of Matrices

**Figure 8:**
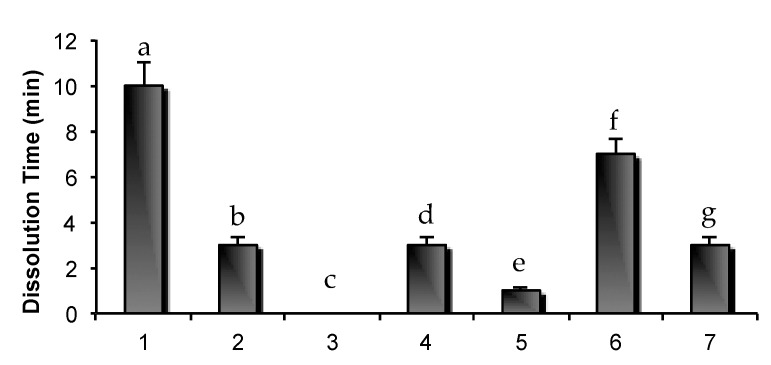
Dissolution times of the different matrices of the experimental design (mean values ± s.e.; *n* = 3). One-way ANOVA, multiple range test (*p* < 0.05): a vs. b/c/d/e/f/g; b vs. e/f; c vs. d/e/f/g; d vs. e/f; e vs. f/g; f vs. g.

In Figure 8, the dissolution times of the different matrices of the experimental design are reported. The matrix based only on GLY 5% (*w*/*w*) immediately dissolved upon contact with buffer solution pH 4.5, while the matrix based only on PVP 3% (*w*/*w*) is characterized by the highest dissolution time (10 min). Considering the two PVP binary mixtures (four and five), the one containing GLY (matrix five) was characterized by the lowest dissolution time.

A correlation could be observed between the mechanical properties (Figure 7) and dissolution time (Figure 8); matrices characterized by the lowest mechanical resistance showed the lowest values of dissolution time.

#### 2.3.3. Experimental Design

**Figure 9:**
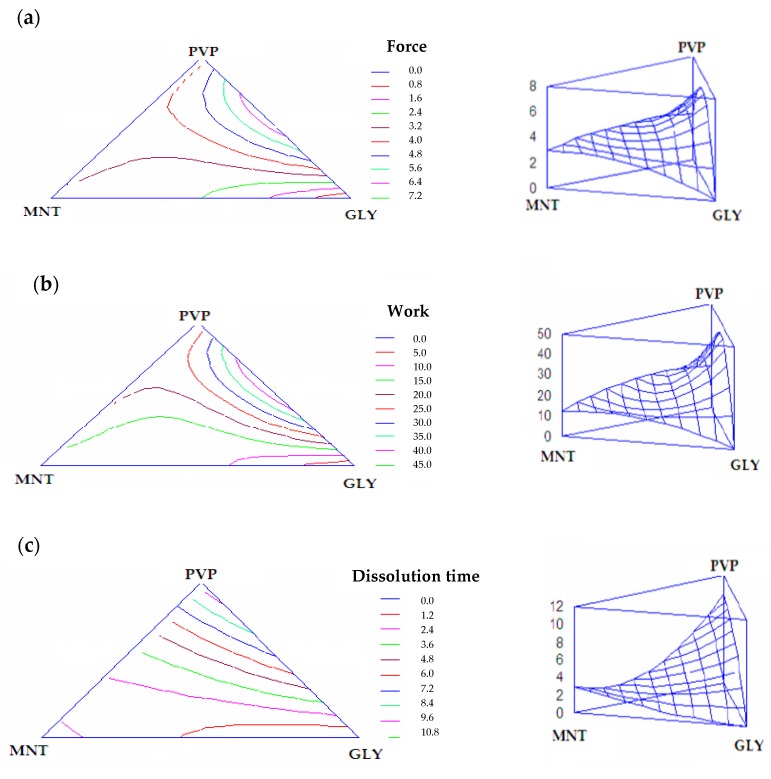
Contour plots (in bidimensional and tridimensional projections) drawn according to model for each response variable: (**a**) force of penetration (N); (**b**) work of penetration (N.mm); (**c**) dissolution time (min).

The best fitting model for all the response variables was found to be a special cubic model. Figure 9 shows the contour plots (in bidimensional projection) drawn according to this model for each response variable. The lines in each plot represent the matrix compositions for which the same response value is predicted by the model. The individual contour plots were subsequently superimposed to identify the region of the factor space fulfilling the following constraints: penetration force > 5 N; penetration work > 35 mN·mm; dissolution time < 6 min. These constraints were chosen bearing in mind that the matrices should possess not only an optimal mechanical resistance, but also a dissolution time compatible with their use. The matrix of optimal composition was PVP 1.3% (*w*/*w*) and GLY 2.8% (*w*/*w*).

The results obtained from the experimental characterization of the mechanical and dissolution properties of the optimized formulation are the following (mean values ± s.d.; *n* = 3): penetration force = 7 ± 1 N; penetration work = 46 ± 2 N.mm; dissolution time = 5 ± 0.5 min. In all cases, the experimental results lie inside the confidence interval of the relevant values predicted by the model, to indicate its predictive power.

#### 2.3.4. NP Loading and Release Properties of Matrices

In Table 4, the size and P.I. of NPs suspended in excipient solution employed for matrix preparation are reported. It can be observed that the maximum NP loading (calculated on matrix dry weight) is equal to 25% *w*/*w*. Nevertheless, for such a concentration, NPs are characterized by a size and P.I. higher than those observed for NPs just prepared (226 ± 7 nm and 0.25 ± 0.030, respectively), indicating that aggregation occurs. The maximum NP loading associated with optimal dispersion properties is 14%. Matrices prepared from 14% *w*/*w* NP excipient suspension were subjected to a dissolution test in acetate buffer (pH 4.5). The dissolution time was 4.8 ± 0.3 min. The comparison of such results with those obtained for the unloaded matrix (5.0 ± 0.3 min) reveals that the presence of NPs does not significantly affect matrix dissolution time. Upon matrix dissolution, the NP size and P.I. values were unchanged with respect to the starting NPs.

## 3. Materials and Methods

### 3.1. Materials

Amoxicillin trihydrate (AX) (IMS, Milan, Italy), ascorbic acid (AA) (Sigma-Aldrich, Milan, Italy), chitosan high molecular weight (CS) (DD 91% (1568), Giusto Faravelli, Milan, Italy); D-Mannitol (MNT) (Sigma Chimica, Milan, Italy); glycin (GLY) (Sigma Chimica, Milan, Italy); pentasodium tripolyphosphate (TPP) (Sigma-Chemicals Co., St. Louis, MO, USA), polyvinylpirrolidone (PVP) (Kollidon^®^90, BASF, Ludwigshafen, Germany) were used.

### 3.2. Preparation of Chitosan Nanoparticles

An exact amount of ascorbic acid (AA) (to obtain a 1:1 molar ratio with chitosan (CS) deacetylated amino groups) was dissolved in distilled water under magnetic stirring for 10 min. Afterwards, chitosan (CS) was added to obtain a 0.1% (*w*/*w*) solution of chitosan ascorbate (CSA) under magnetic stirring for 48 h. Aqueous pentasodium tripolyphosphate (TPP) solutions at three different concentrations (0.96 mg/mL, 0.83 mg/mL, 0.69 mg/mL) were prepared. NPs were manufactured according to the mild operative procedure developed by Calvo et al. [31], based on the ionotropic gelation of CSA and TPP. In detail, 1.2 mL of TPP solution were added to 3 mL of CSA solution under magnetic stirring (200 rpm) at room temperature for 10 min. As the TPP concentration decreased, NPs with increasing CSA/TPP *w*/*w* ratios (2.6:1, 3:1 and 3.6:1) were obtained. In the case of AX-loaded NPs, AX was dissolved in the TPP solution under gentle magnetic stirring for 30 min. Subsequently, the TPP solution (1.2 mL) was added to the CSA solution (3 mL). Different amounts of AX were considered in order to obtain the following CSA/AX *w*/*w* ratios: 3:1, 4:1 and 6:1.

### 3.3. Characterization of Unloaded NPs (Blank)

#### 3.3.1. Particle Size

The NP size and polydispersion index were measured at 25 °C by Photon Correlation Spectroscopy (Beckman Coulter N5; Instrumentation, Beckman Coulter, Milan, Italy), at an angle of 90°, after dilution in bidistilled and filtered (0.45 μm, Millipore, Vimodrone, Italy) water.

#### 3.3.2. Production Yield

The production yield was determined by a gravimetric test. In detail, NP suspension was centrifuged at 22,000 rpm for 45 min at 25 °C in Eppendorf vials containing 300 μL of glycerol; then, the supernatant was removed. The precipitated NPs were freeze-dried (Heto Dryer, Analytica De Mori, Milano, I) for 24 h and subsequently weighed (*n* = 3). Production yield (Y), expressed as a percentage, was calculated according to the following Equation (1):

Y(%) = (NP weight/total solid weight) × 100(1)

where total solid weight = total weight of reagents (CS + AA + TPP) used for NP production.

### 3.4. Characterization of AX-Loaded NPs

#### 3.4.1. Size and Production Yield

The size, polydispersion index and production yield of the loaded NPs were obtained as previously described (see Section 3.3.1 and Section 3.3.2). Particle size and polydispersion index of loaded NPs were measured immediately after preparation and after storage for 24 h, 48 h and seven days at 4 °C. zeta potential was measured on freshly prepared samples by laser Doppler anemometry using a Zetasizer 3000 HS (Malvern Instruments, Malvern, UK).

#### 3.4.2. AX Loading Capacity and Association Efficiency

Loaded NPs were separated from the aqueous medium by ultracentrifugation at 22,000 rpm at 45 °C for 45 min in Eppendorf vials on a bed of glycerol (300 µL). The AX amount in the collected supernatant was dosed by a reversed-phase (RP) HPLC method (Perkin Elmer series 200, Monza, Italy). In detail, a C18 RP column was used as the stationary phase and phosphate buffer pH 5:acetonitrile (96:4 *v*/*v*) was employed as the mobile phase. The drug was spectrophotometrically detected at a wavelength of 230 nm. The loading capacity (LC%) and association efficiency (AE%) were calculated according to the following equations (Equations (2) and (3)):

LC% = (Dt − Ds/NP weight) × 100(2)

AE% = (Dt − Ds/Dt) × 100(3)

where Dt = total drug amount (i.e., AX amount (mg) added to TPP solution); Ds = free drug amount (i.e., AX amount (mg) dosed in supernatant).

#### 3.4.3. Mucoadhesion Properties

The mucoadhesion properties of NPs based on 2.6:1 *w*/*w* CSA/TPP and 4:1 *w*/*w* CSA/AX ratios were assessed by means of turbidimetric measurements [16,17]. In particular, gastric mucin type III (Sigma, Milan, Italy) was dispersed in acetate buffer (pH 4.5) under mild stirring at room temperature at the following concentrations: 0.025, 0.05, 0.1, 0.25, 0.5, 0.75, 1, 1.5% (*w*/*v*). Mixtures of 1:1 (*v*/*v*) of NP suspension with mucin dispersion were prepared. NP–mucin mixtures were maintained at rest for 30 min at room temperature before further characterization.

The turbidimetric measurements were carried out using a spectrophotometer (UV-vis Lamba 25, Perkin Elmer, Milan, Italy) at λ = 500 nm (according to Eur. Pharm.). The turbidimetry values of NP–mucin mixtures were compared with those of the dispersions containing the same concentrations of mucin as in the mixtures.

#### 3.4.4. Antimicrobial Activity Measurements

Measurements were carried out on blank NPs prepared according to a 2.6:1 *w*/*w* CSA/TPP ratio, and on loaded NPs prepared according to a 2.6:1 *w*/*w* CSA/TPP ratio and 4:1 *w*/*w* CSA/AX. Both blank and loaded NPs were centrifuged, as reported in Section 2.3.2, and then suspended in distilled water. The antimicrobial activity of loaded NPs was compared with the activity of an aqueous solution with the same concentration of chitosan as in the NP suspension. The antimicrobial activity was evaluated against two standard strains, *Enterococcus hirae* ATCC 10541 and *Streptococcus pyogenes* ATCC 19615, as the minimum inhibitory concentration (MIC). The MIC was defined as the lowest sample concentration (% *w*/*v*) that completely inhibited bacteria growth after incubation for 24 h at 37 °C.

The MIC values were determined according to the standard broth macrodilution method described by the NCCLS (National Committee for Clinical Laboratory Standards) procedure, performed in Mueller Hinton Broth (Oxoid, Basingstoke, UK) with an average inoculum of 1 × 10^7^ CFU/mL.

Before testing, bacteria were grown overnight in Tryptone Soya Broth (Oxoid, Basingstoke, UK) at 37 °C. The cell suspension was centrifuged at 2000 rpm for 20 min to separate cells from broth and then suspended in phosphate buffer saline (PBS) (Sigma-Aldrich, Milan, Italy) at pH 7.3. The suspension was diluted to adjust the number of cells to 1 × 10^7^–1 × 10^8^ CFU/mL. An aliquot of sample was added to the microbial suspensions. For each tested strain, a bacteria suspension was prepared in PBS without sample and used as control. The results are expressed as % MIC values by normalizing the MIC values obtained for the samples to that measured for the control.

#### 3.4.5. Proliferation Assay

Normal human dermal fibroblasts (NHDF) (PromoCell GmbH, Heidelberg, Germany) were used. Cells between the second and fifth passage were used for the experiments. Dulbecco’s modified Eagle medium (DMEM, Sigma, Milan, Italy), supplemented with 10% foetal calf serum (FCS, Sigma, Milan, Italy), 200 U/mL penicillin and 0.2 mg/mL streptomycin (Sigma, Milan, Italy) was used as the growth medium. The cells were incubated at 37 °C with 95% air and 5% CO_2_ atmosphere. All the operations required for the cell culture were carried out in a vertical laminar air flow hood (Ergosafe Space 2, PBI International, Milan, Italy).

The cell proliferation was evaluated to assess the capability of the NPs to improve NHDF fibroblast growth by using a neutral red assay. The samples tested were blank NPs prepared according to 2.6:1 (*w*/*w*) CSA/TPP ratio; loaded NPs prepared according to 2.6:1 CSA/TPP (*w*/*w*) ratio and 4:1 (*w*/*w*) CS/AX ratio; polymer aqueous solution (CSA 0.135% *w*/*w*). Each sample was diluted 1:10 *w*/*w* in medium with serum (M (w/s)). Complete medium (CM) and a Triton solution (Triton X-100, Biochemika Fluka, Milano, I) were used respectively as positive and negative controls. For each sample, 8 wells were considered.

In detail, 20 μL of cell suspension (7.5 × 10^4^ cells) together with 200 μL of each sample, previously diluted, were seeded in each well (0.34 cm^2^ area) of a 96-well plate and incubated for 24 h. Afterwards, the samples were removed and 200 μL of neutral red (NR) (Tox Kit 4, Sigma-Aldrich, Milan, Italy) solution in M w/s (0.33 mg/mL) were added to each well and incubated for 2 h at 37 °C. Then, adherent cells were washed with phosphate buffer solution (PBS), fixed by using a solution of CaCl_2_ 1% (*w*/*v*) and formaldehyde 0.5% (*w*/*v*) and, subsequently, added with 200 μL of a solubilisation solution (CH_3_COOH 11% *w*/*v* in ethanol 50% *v*/*v*), which was able to induce the disruption of cellular and lysosomal membranes in order to release the NR internalized only by vital and undamaged cells. Subsequently, the absorbance was read at a wavelength of 490 nm, with a wavelength reference of 655 nm, by an ELISA microplate absorbance reader (Bio-Rad Laboratories S.r.l., Segrate–Milan, Italy).

#### 3.4.6. In Vitro Wound Healing Assay

The in vitro wound healing assay was performed using Petri μ-Dish (Ibidi, Giardini, Milan, Italy) in which an insert was enclosed. This insert was composed of two chambers with a growth area of 0.22 cm^2^, divided by a septum that produced a cell free gap with a width of 500 ± 50 µm.

Fibroblasts were seeded in each chamber at 10^5^ cells/cm^2^ and grown at a confluence in standard conditions, as previously described. After 24 h, cells reached confluence and the insert was removed revealing two cell monolayers divided by the prefixed gap. Cell substrates were put in contact with 200 μL of each sample diluted with 800 μL of medium without serum. The following samples were considered: blank NPs prepared according to 2.6:1 (*w*/*w*) CSA/TPP ratio; loaded NPs prepared according to 2.6:1 CSA/TPP (*w*/*w*) ratio and 4:1 (*w*/*w*) CS/AX ratio; polymer aqueous solution (CSA 0.135%); medium without serum (Mw/s). At prefixed times (0 and 24 h), microphotographs were taken to evaluate cell migration and growth in the gap.

### 3.5. Preparation of Fast Dissolving Matrices

**Figure 10:**
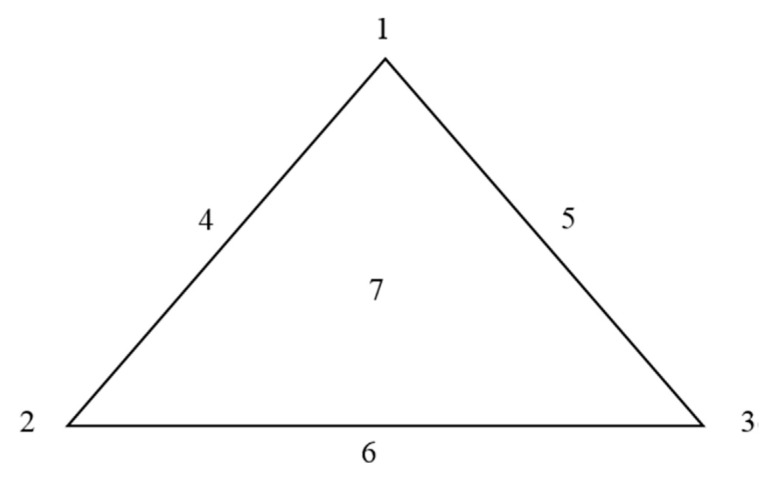
Factor space and experimenting points of a simplex centroid design.

The choice of the optimal matrix composition was carried out by a suitable experimental design (DoE): the simplex centroid design for three components. Three aqueous stock solutions of PVP 3% *w*/*w*, MNT 10% *w*/*w*, GLY 5% *w*/*w* were considered. The three stock solutions were used as such or mixed in predetermined *w*/*w* ratios to obtain the significant points (7) of the simplex centroid design, corresponding to the characteristic points of a Sheffé triangle (Figure 10). The fractional mixture composition is reported in Table 5. Points one, two and three correspond to the three stock solutions and are the vertices of the triangle. Points four, five and six were obtained by mixing in 1:1 *w*/*w* ratio the stock solutions. The centroid (point seven) corresponds to the ternary mixture (1/3 *w*/*w* of each stock solution).

#### 3.5.1. Matrix Preparation

2 g of each solution/mixture were poured into an Eppendorf vial, froze for at least 12 h at −20 °C and then freeze-dried (Heto Drier, Analitica De Mori, Milan, Italy) for 24 h.

#### 3.5.2. Assessment of Matrix Mechanical Properties

Matrices were subjected to tensile measurements by means of a TA.XTplus apparatus (Stable Micro Systems, Surrey, UK), equipped with a 1 kg load cell and a P/50 measuring system, consisting of a cylindrical probe (∅ 50 mm). Matrices placed in plastic containers used for their preparation were put on the base of the instrument and kept vertical; the probe was lowered at a rate of 10 mm/s inside the sample up to a fixed depth equal to 10 mm. The parameters measured were penetration force (F) and penetration work (L), calculated as the area under the F vs. distance curve. For each matrix, three measurements were performed (*n* = 3).

#### 3.5.3. Assessment of Matrix Dissolution Time

Each matrix was placed in a glass tube containing 3 mL of acetate buffer pH 4.5 to simulate the vaginal environment. The glass tube was thermo-stated at 37 °C and agitated at 50 rpm by means of a thermostatic bath (Falc Instruments Mod. WB-MF24, Treviglio, Italy). For each matrix the time necessary to obtain a complete dissolution was measured; three measurements were performed (*n* = 3).

#### 3.5.4. Optimization Procedure

The response variables of the experimental design were penetration force (mN), penetration work (mN·mm) and dissolution time (min). Each response variable could be related to the mixture composition by means of a suitable mathematical model that adequately described the relation. Model selection was performed using a statistical software package (Statgraphics 5.0, Statistical Graphics, Rockville, MD, USA). Experimental data were treated according to a multiple linear regression analysis testing a series of models including linear, quadratic, and special cubic [32]. The best fit model was chosen on the basis of statistical parameters, such as the F-ratio for significance of regression and the adjusted correlation coefficient for the goodness-of-fit of the model.

The matrix of optimized composition, chosen on the basis of the result of the experimental design, was prepared by freeze-drying (see Section 3.5) and subjected to the same characterization previously carried out on the seven formulations of the Sheffè triangle. The experimental results were compared with those provided by the model to confirm its predictive power.

### 3.6. Preparation of Matrix Loaded with NPs

AX-loaded NPs were isolated by ultracentrifugation as indicated above (see Section 2.2) and suspended in 2 g of PVP/GLY/MNT mixture of optimized composition. Samples, placed in Eppendorf vials, were frozen for at least 12 h at −20 °C and then freeze-dried for 24 h (Heto Dryer, Analytica De Mori, Milan, Italy).

### 3.7. Matrix Release Properties

NP-loaded matrices were placed in a glass tube containing 3 mL of acetate buffer pH 4.5 to simulate the vaginal environment. The glass tube was thermostated at 37 °C and agitated at 50 rpm by means of a thermostatic bath (Falc Instruments Mod. WB-MF24, Treviglio, Italy).

After complete matrix dissolution, 200 μL of the dissolution media were collected, diluted in bidistilled water and analyzed at 25 °C by Photon Correlation Spectroscopy (Beckman Coulter N5; Instrumentation, Beckman Coulter, Milan, Italy) at an angle of 90°.

### 3.8. Statistical Analysis

Whenever possible, experimental values collected from various types of measures were subjected to statistical analysis, carried out by means of the statistical package Statgraphics Plus (Statgraphics 5.0, Statistical Graphics Co., Rockville, MD, USA). In particular, an ANOVA one-way multiple range test was used.

## 4. Conclusions

In the present study, a fast dissolving polymeric matrix loaded with CSA NPs was developed for the vaginal administration of antibiotic drugs. Upon contact with vaginal fluids, the matrix should dissolve and release the loaded NPs. CSA NPs should ensure drug spreading on the vaginal mucosa and enhance its permanence in the vaginal cavity.

The optimal weight ratios between CSA and TPP and CSA and AX were identified on the basis of NP size, polydispersion index and production yield. Loaded NPs proved a good physical stability in aqueous suspension, suggesting optimal dispersion properties, which guarantee homogenous incorporation into the polymeric matrix. Preliminary studies of NP antimicrobial activity were performed on two different microorganism strains, generally responsible for vaginal infections. The blank nanoparticles proved more effective in inhibiting the bacteria growth than a chitosan solution containing the same polymer concentration. Both blank and loaded NPs were able to enhance fibroblast proliferation and the wound healing process to the same extent of the corresponding chitosan aqueous solution, proving that neither the NP preparation method nor the presence of the drug alter the intrinsic bioactive properties of chitosan.

Subsequently, a polymeric matrix in which NPs were loaded was developed and optimized. The experimental design used (simplex centroid design) allowed the identification of the best matrix composition.

The comparison of physical properties (size and polydispersion index) of NPs, fresh and after matrix dissolution, allowed the identification of the maximum NP concentration to be loaded in the matrix. Moreover, it was demonstrated that NPs maintained their size upon release in simulated vaginal fluid from the optimized polymeric matrix.

In conclusion, CSA NPs loaded in a fast-dissolving matrix represent a promising candidate for the treatment of vaginal atrophy.

## Figures and Tables

**Table 1:** Size, polydispersion index (P.I.) and yield (%) values of blank nanoparticles (NPs) prepared according to different chitosan ascorbate/tripolyphosphate (CSA/TPP) *w*/*w* ratios (mean values ± s.e.; *n* = 3).

CSA/TPP *w*/*w* Ratio	Size (nm)	P.I.	Yield (%)
**2.6:1**	215 ± 3	0.10 ± 0.02	30 ± 1
**3:1**	236 ± 6	0.22 ± 0.03	22 ± 0.9
**3.6:1**	258 ± 6	0.28 ± 0.02	12 ± 1

**Table 2:** Size, polydispersion index (P.I.) and yield (%) values ofamoxicillin (AX)-loaded NPs prepared according to different CSA/TPP *w*/*w* ratio and CSA/AX *w*/*w* ratios (mean values ± s.e.; *n* = 3).

CSA/TPP *w*/*w* Ratio	Size (nm)	P.I.	Yield (%)
CSA/AX 3:1 *w*/*w* ratio			
2.6:1	232 ± 3	0.403 ± 0.080	43 ± 0.3
3:1	210 ± 3	0.415 ± 0.010	63 ± 0.1
3.6:1	256 ± 1	0.567 ± 0.060	43 ± 0.3
CSA/AX 4:1 *w*/*w* ratio			
2.6:1	226 ± 7	0.251 ± 0.030	68 ± 0.2
3:1	215 ± 1	0.266 ± 0.020	74 ± 0.7
3.6:1	268 ± 5	0.592 ± 0.040	55 ± 0.2
CSA/AX 6:1 *w*/*w* ratio			
2.6:1	234 ± 6	0.461 ± 0.040	73 ± 0.2
3:1	216 ± 1	0.354 ± 0.004	83 ± 0.3
3.6:1	228 ± 4	0.615 ± 0.040	64 ± 0.4

**Table 3:** Antimicrobial properties (expressed as minimum inhibitory concentration (MIC) values) of blank and AX-loaded NPs in comparison with a chitosan ascorbate (CSA) aqueous solution prepared at the same concentration employed for NP preparation.

Microrganism	CSA Solution	Blank NPs	Loaded NPs
*E. hirae*	0.12%	0.03%	0.004%
*S. pyogenes*	0.06%	0.015%	0.0001%

MIC values are expressed as % *w*/*v* CSA.

**Table 4:** Size and P.I. of NPs suspended into the excipient solution and upon matrix dissolution (mean values ± s.e.; *n* = 3).

	NPs Suspended in Excipient Solution		NPs Upon Matrix Dissolution	
% NPs Loaded into Matrix	Size (nm)	P.I.	Size (nm)	P.I.
8	220 ± 8	0.51 ± 0.03	-	-
14	222 ± 7	0.44 ± 0.06	225 ± 5	0.58 ± 0.07
25	338 ± 25	1.4 ± 0.07	-	-
40	- *	- *	-	-

* Not suspendible.

**Table 5:** Fractional mixture composition corresponding to the characteristic points of a Sheffè triangle.

Samples	X1 (PVP)	X2 (MNT)	X3 (GLY)
1	1	0	0
2	0	1	0
3	0	0	1
4	1/2	1/2	0
5	1/2	0	1/2
6	0	1/2	1/2
7	1/3	1/3	1/3
